# Hypoxia-induced PLOD1 overexpression contributes to the malignant phenotype of glioblastoma via NF-κB signaling

**DOI:** 10.1038/s41388-020-01635-y

**Published:** 2021-01-08

**Authors:** Zhenlin Wang, Yuping Shi, Chenting Ying, Yang Jiang, Jiangfeng Hu

**Affiliations:** 1 Department of Neurosurgery, Shanghai General Hospital, Shanghai Jiao Tong University School of Medicine, 100 Haining Road, Shanghai, China; 2 Department of Nephrology, Shanghai TongRen Hospital, Shanghai Jiao Tong University School of Medicine, 1111 Xianxia Road, Shanghai, China; 3 Department of Orthopedics, Shanghai General Hospital, Shanghai Jiao Tong University School of Medicine, 100 Haining Road, Shanghai, China; 4 Department of Neurosurgery, Shanghai Tenth People’s Hospital, Tongji University School of Medicine, Shanghai, 200072 China; 5 Department of Gastroenterology, Shanghai General Hospital, Shanghai Jiao Tong University School of Medicine, 100 Haining Road, Shanghai, China

**Keywords:** Cancer stem cells, CNS cancer

## Abstract

Procollagen lysyl hydroxylase 1 (PLOD1) is highly expressed in malignant tumors such as esophageal squamous cell carcinoma, gastric cancer, and colorectal cancer. Bioinformatics analysis revealed that PLOD1 is associated with the progression of GBM, particularly the most malignant mesenchymal subtype (MES). Moreover, in the TCGA and CGGA datasets, the mean survival time of patients with high PLOD1 expression was significantly shorter than that of patients with low expression. The clinical samples confirmed this result. Therefore, we aimed to investigate the effect of PLOD1 on the development of mesenchymal GBM in vitro and in vivo and its possible mechanisms. Molecular experiments were conducted on the patient-derived glioma stem cells and found that PLOD1 expressed higher in tumor tissues and cancer cell lines of patients with GBM, especially in the MES. PLOD1 also enhanced tumor viability, proliferation, migration, and promoted MES transition while inhibited apoptosis. Tumor xenograft results also indicated that PLOD1 overexpression significantly promotes malignant behavior of tumors. Mechanistically, bioinformatics analysis further revealed that PLOD1 expression was closely associated with the NF-κB signaling pathway. Besides, we also found that hypoxic environments also enhanced the tumor-promoting effects of PLOD1. In conclusion, overexpression of PLOD1 may be an important factor in the enhanced invasiveness and MES transition of GBM. Thus, PLOD1 is a potential treatment target for mesenchymal GBM or even all GBM.

## Introduction

Glioblastoma multiforme (GBM) is the most common primary brain malignancy with the worst prognosis. Its average survival time is less than 15 months [1, 2]. GBM is diagnosed as a grade IV in the WHO classification criteria [1, 3]. Verhaak et al. proposed four molecular subtypes of GBM based on the different gene expression, including proneural, classical, and mesenchymal subtype [4, 5]. Molecular subtyping has been regarded as a promising strategy to predict GBM evolution, common disease pathways, and rational treatment options [6]. Patients with MES GBM have the shortest median survival and the worst response to radiotherapy and chemotherapy [2, 7]. Moreover, studies have found that the natural course of low-grade gliomas and GBM is often accompanied by MES transition during treatment, promoting tumor proliferation, invasion, and treatment tolerance [7–9]. A subpopulation of cells with stem cell-like properties, known as glioma initiating cells or glioma stem cells (GSCs), exists in GBM and plays the potential roles for multi-lineage differentiation and self-renewal that can lead to tumorigenesis, metastasis, recurrence, and radiotherapy and chemotherapy resistance [10–13]. Therefore, identifying genes associated with growth, invasion, and MES transition of GSCs in mesenchymal GBM has significant implications for glioma clinical treatment and basic research.

The procollagen lysyl hydroxylase (PLOD) genes include PLOD1, PLOD2, and PLOD3, which encode procollagen lysine, 2-oxoglutarate 5-dioxygenase that regulates collagen synthesis, cross-linking, and deposition [14]. Collagen is an important component that constitutes the extracellular matrix (ECM) of tumors, and cross-linking of collagen in ECM enhances the hardness of tumor tissue and promotes proliferation, migration, invasion, and adhesion of tumor cells [15, 16]. Mutations and overexpression of PLOD promote the occurrence and metastasis of multiple malignancies [17]. The PLOD1 gene is located on chromosome 1p36.2–36.3 and contains 19 exons [18]. Many studies have shown that PLOD1 is involved in the occurrence and development of multiple tumors. PLOD1 overexpression was observed in bladder cancer and intensively correlated with poor patient prognosis [19]. Overexpression PLOD1 promoted proliferation of breast cancer and lymph node and lung metastasis through regulating collagen cross-linking [20]. Mutations or overexpression of PLOD1 were also detected in esophageal squamous cell carcinoma, gastric cancer, and colorectal cancer and associated with shorter patient survival [21, 22]. However, available research does not address the relationship between PLOD1 and GBM and whether PLOD1 is involved in the occurrence, development, and malignant phenotype of GBM.

Using bioinformatics analysis of TCGA and CGGA datasets, we found that PLOD1 was highly expressed in GBM, especially in the mesenchymal subtype, and was linked to patients’ poor prognosis. Further GSEA analysis revealed that PLOD1 is strongly associated with the nuclear factor-κB (NF-κB) signaling pathway. The NF-κB signaling pathway regulates the tumor microenvironment of GSCs, promotes vascular regeneration, and improves treatment tolerance of tumor cells [23, 24]. Meanwhile, NF-κB is a dependent signaling pathway in the MES transition of GSCs that upregulates CD44 expression, promoting the malignant transformation (MES transition) of GSCs [12]. NF-κB activation is strongly associated with shorter survival in patients with GBM [12, 25]. Besides, it has been shown that hypoxic environment enhances PLOD1 and PLOD2 expression [20, 26], and hypoxia, an important feature of GBM cells, has a role in promoting tumor cell growth, invasion, and MES transition, etc. [27]. We further performed both in vitro and vivo experiments to investigate the effects of PLOD1 on GSCs proliferation, invasion, anti-apoptosis, and MES transition in GBM under hypoxic conditions, demonstrating that PLOD1 can be a potential therapeutic target for GBM, especially mesenchymal GBM.

## Results

### PLOD1 is upregulated in MES GBM and inversely correlated with survival

**Fig. 1:**
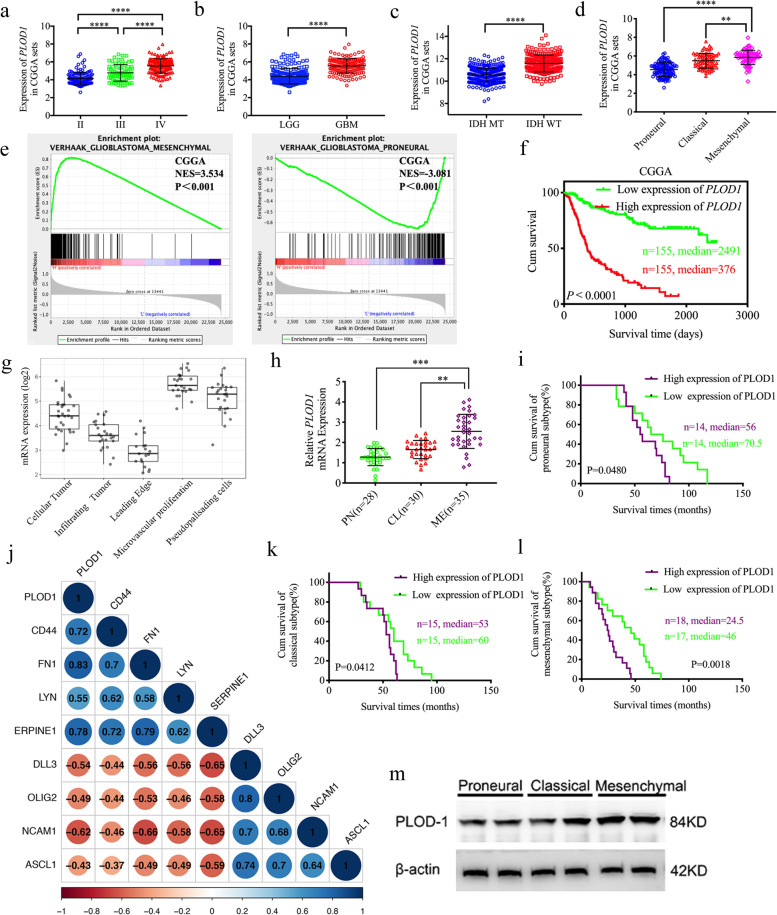
PLOD1 is expressed at a higher level in the mesenchymal GBM and is associated with poor patient survival. **a**–**d** The mRNA expression of PLOD1 is shown according to WHO grades, GBM or LGG, IDH status, and the molecular subtypes in the CGGA datasets. **e** Gene set enrichment analysis (GSEA) indicates that mesenchymal subtype enrichment occurs when PLOD1 is highly expressed, while proneural subtypes are enriched when PLOD1 is lowly expressed in the CGGA datasets. **f** Kaplan–Meier analysis of patients with GBM with high PLOD1 expression versus low PLOD1 expression in the CGGA datasets. **g** Ivy atlas of PLOD1 expressed higher in the core than the tumor’s outer margin. **h** PLOD1 is expressed at higher levels in patients with GBM with mesenchymal subtype than another two subtypes as measured by qPCR. **i**–**l** Kaplan–Meier analysis of the three molecular subtypes of patients with GBM in terms of higher PLOD1 expression versus low PLOD1 expression. **j** The expression of PLOD1 is positively correlated with the expression of mesenchymal-related genes, while is opposite to the proneural related genes in the CGGA datasets. **m** Western blotting showed PLOD1 expression in clinical GBM specimens of different molecular subtypes. All data are shown as the mean ± SD (three independent experiments). *P < 0.05; **P < 0.01; ***P < 0.001.

We first examined PLOD1 mRNA expression in public available glioma dataset CGGA and found that PLOD1 expression was highest in grade IV glioma and lowest in grade II (Fig. 1a). Expression levels were significantly higher in GBM than in low-grade glioma (Fig. 1b). And IDH wild-type PLOD1 expression levels were higher than IDH mutant in CGGA datasets (Fig. 1c). We also analyzed the correlation between the expression of PLOD1 and the molecular subtype of GBM. We showed that the level of PLOD1 expression was significantly higher in the mesenchymal subtype than in the proneural, neuronal, and classic subtype (Fig. 1d). Subsequently, we performed a GSEA analysis of the relationship between PLOD1 and mesenchymal or proneural subtype based on the CGGA dataset. The results showed that enrichment of the mesenchymal subtype was present at high PLOD1 expression group, whereas enrichment of the proneural subtype was present at low PLOD1 expression group (Fig. 1e). At the same time, we found a significant positive correlation between the expression of PLOD1 and the expression of mesenchymal subtype-related genes (CD44, FN1, LYN, SERPINE1) in the CGGA dataset, whereas genes associated with the proneural subtype (DLL3, OLIG2, NCAM1, ASCL1) were negatively correlated (Fig. 1j). Besides, PLOD1 expressed higher in the core than the tumor’s outer margin according to the Ivy atlas (Fig. 1g). In addition, we performed Kaplan–Meier survival analysis for the prognostic significance of PLOD1 expression in patients with GBM, and the average survival time of patients with high PLOD1 expression was significantly lower in the CGGA dataset than in patients with low expression (Fig. 1f). We also detected the expression of PLOD1 in the TCGA dataset and the same results were obtained in Supplementary Fig. S1.

We then detected the PLOD1 expression from a total of 93 patients with GBM by both qPCR and western blotting and confirmed the findings obtained by bioinformatics analysis. The highest PLOD1 expression levels were found to be presented in the mesenchymal subtype, while there was no statistically significant difference among the remaining two subtypes (Fig. 1h, m). In addition, we performed Kaplan–Meier survival analysis on patients (qPCR quantification, Cutoff: median), and the results showed that the median survival of mesenchymal patients with higher PLOD1 expression was significantly lower than that of patients with low expression, while there was no survival significance in another three subtypes (Fig. 1i, k, l).

We further extracted patient-derived GSCs from fresh clinical GBM specimens for culture. Supplementary Figure S2a showed hematoxylin and eosin (H&E) staining of the tumor cells of the original patients. Six GSCs with the best growth status were selected for molecular typing. By flow analysis, three strains of PN03-GSC, PN04-GSC, and PN09-GSC were positive for CD133, a proneural marker, by more than 80%, and three strains of MES02-GSC, MES06-GSC, and MES13-GSC were positive for CD44, a mesenchymal marker, by more than 80% (Supplementary Fig. S2b) [28]. Immunofluorescence staining confirmed the presence of CD133+, CD44+, and nestin+ expression in isolated neurospheres (Supplementary Fig. S2c). Supplementary Figure S2d demonstrates the multi-lineage differentiation ability of GSCs.

We then measured PLOD1 expression in the above GSCs by qPCR and western blotting. It was seen that PLOD1 expressed higher in GSCs than normal human astrocyte (NHA) (Supplementary Fig. S2e), and the expression of PLOD1 in the three strains of mesenchymal subtype GSCs was even higher than that of the proneural subtype (Supplementary Fig. S2e, f). Moreover, PLOD1 was positively correlated with the mesenchymal marker genes YKL40 and CD44 and negatively correlated with the proneural marker gene OLIG2 (Supplementary Fig. S2f). We further classified three mesenchymal cell lines MES02-GSC, MES06-GSC, and MES13-GSC into CD44^high^ and CD44^low^ subpopulations by flow sorting, higher PLOD1 expression (Supplementary Fig. S2h, i) was detected in CD44^high^ by qPCR and western blotting. In summary, the expression of PLOD1 in mesenchymal GBM was significantly higher than other subtypes, and the higher expression of PLOD1 predicted a significantly poorer prognosis in patients with GBM, suggesting that PLOD1 may play an important role in mesenchymal GBM or even in all GBM.

### PLOD1 knockout inhibits MES GSC-enriched tumor sphere growth and invasion in vitro

**Fig. 2:**
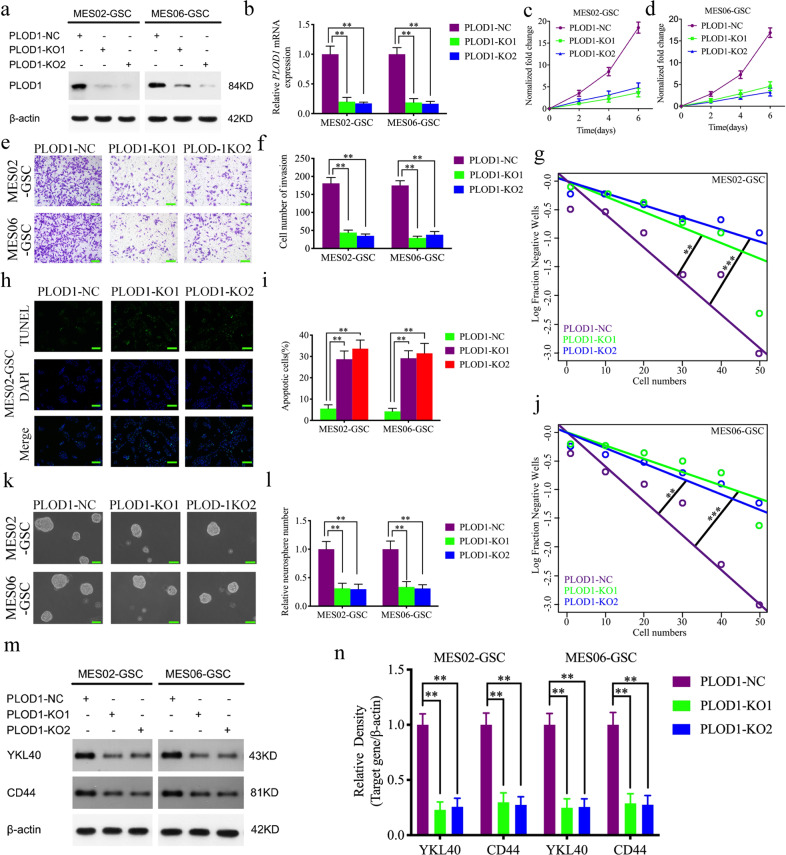
PLOD1 knockout inhibits MES GSCs’ malignant behaviors in vitro. **a**, **b** The expression of PLOD1 in MES02-GSC and MES06-GSC after transfection of PLOD1-KO1, PLOD1-KO2, or negative control as measured by western blotting and qPCR. **c**, **d** PLOD1 knockout significantly reduced the proliferation of MES02-GSC and MES06-GSC in MTS assays. **e**, **f** Transwell assays showed the invasion of MES02-GSC and MES06-GSC after PLOD1 knockout. Scale bar = 50 μm. **g**–**l** Neurospheres formation assays and extreme limit dilution assays showed that the tumor formation rates decreased after PLOD1 knockout in MES02-GSC and MES06-GSC. Scale bar = 20 μm. **h**, **i** PLOD1 knockout can significantly increase the apoptosis rates of MES02-GSC and MES06-GSC as measured by TUNEL assays (terminal deoxynucleotidyl transferase deoxyuridine triphosphate gap end-labeling method). Scale bar = 50 μm. **m**, **n** The protein expression of YKL40 and CD44 in MES02-GSC and MES06-GSC after transfection of PLOD1-KO1, PLOD1-KO2, or negative control as measured by western blotting and gray quantitative analysis. All data are shown as the mean ± SD (three independent experiments). *P < 0.05; **P < 0.01; ***P < 0.001.

To investigate the role of PLOD1 in GSCs proliferation and tumor promotion, Crispr-Cas9-mediated PLOD1 knockout was performed and mesenchymal GSCs MES02-GSC and MES06-GSC was transfected to knockout PLOD1. Western blotting and qPCR were used to confirm the efficiency of PLOD1 knockout in MES02-GSC and MES06-GSC (Fig. 2a, b). First, we used the MTS assays to detect the proliferation activity of tumor cells after PLOD1 knockout, and the absorbance values were significantly lower than those of the control group (Fig. 2c, d), confirming that PLOD1 knockout inhibits the proliferation of GSCs. Subsequently, we examined the effect of PLOD1 knockout on tumor cell invasion by the transwell assays, as shown in Fig. 2e, f, where tumor cells’ invading ability was significantly inhibited after PLOD1 knockout compared to the control group (Fig. 2e, f). In addition, we used TUNEL assays to detect the effect of PLOD1 knockout on apoptosis in GSCs and showed that PLOD1 knockout significantly promoted apoptosis in tumor cells (Fig. 2h, i). Neurosphere formation assays showed the proliferative activity of neurospheres after PLOD1 knockout was much less than that of controls (Fig. 2k, l). The extreme limiting dilution assays also showed a reduction in tumor formation after PLOD1 knockout (Fig. 2g, j and Supplementary Table 1). Finally, we again performed western blotting and found that the expressions of the mesenchymal marker genes CD44 and YKL40 were significantly reduced after PLOD1 knockout in MES02-GSC and MES06-GSC (Fig. 2m, n). The above results indicated that PLOD1 knockout inhibits glioma cell proliferation, invasion, and MES transition of glioma and induces its apoptosis.

### PLOD1 overexpression promotes growth, invasion, and MES transition of GSCs in vitro

To further demonstrate the role of PLOD1 in GSCs, we designed lentiviral-encapsulated PLOD1 overexpression plasmids and transfected with the proneural subtype cell lines PN03-GSC and PN04-GSC, respectively. Both western blotting and qPCR assays confirmed the ideal effect of PLOD1 overexpression (Supplementary Fig. S3a, b). MTS assays showed that the absorbance values were significantly higher than controls (Supplementary Fig. S3c, f), confirming that PLOD1 overexpression promoted the proliferation of GSCs. Transwell assays showed that PLOD1 overexpression markedly promoted tumor cell invasion and increased migration compared with controls (Supplementary Fig. S3d, e). TUNEL assays results showed that PLOD1 overexpression inhibited the apoptotic process (Supplementary Fig. S3g, h). Moreover, neurosphere formation assays showed that the relative numbers of neurospheres were greater than those in the control group after PLOD1 overexpression (Supplementary Fig. S3j, k). We also found that the tumor formation rates increased after PLOD1 overexpression as measured by the extreme limiting dilution assays (Supplementary Fig. S3l, m and Supplementary Table 1). Western blotting demonstrated that the expression of the mesenchymal marker genes CD44 and YKL40 was significantly increased after PLOD1 overexpression (Supplementary Fig. S3i, n). The above results confirm that PLOD1 has a key role in the MES transition and proliferation of tumors.

### PLOD1 regulates the NF-κB signaling pathway in GSCs

**Fig. 3:**
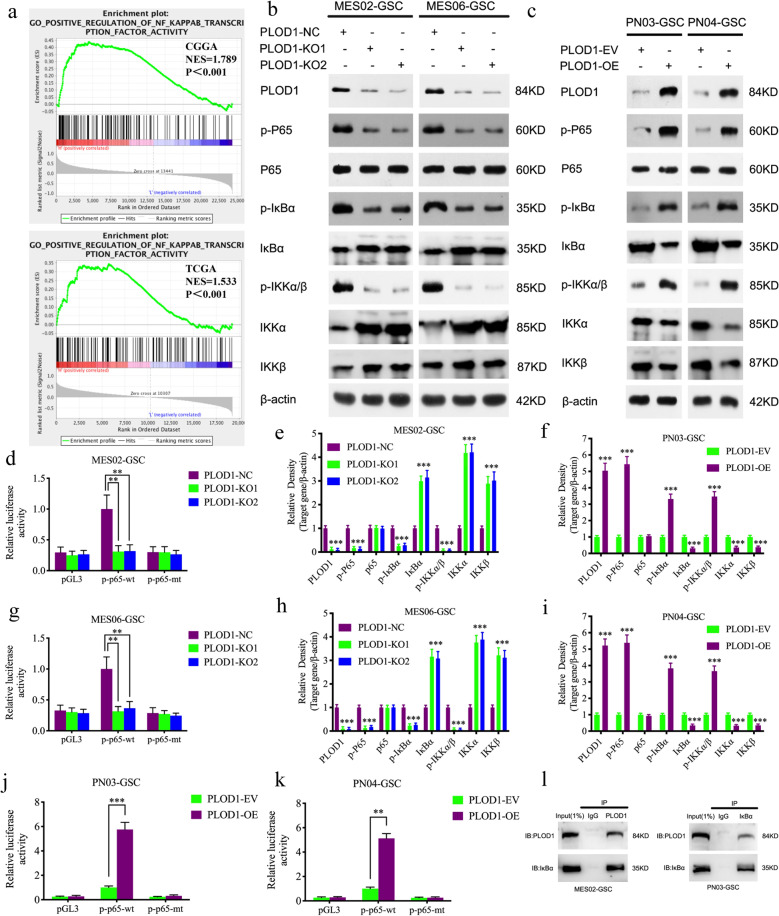
PLOD1 regulates the malignant biological behavior of GBM through the NF-κB pathway. **a** GSEA analysis showed that high expression of PLOD1 was positively correlated with enhanced expression of the NF-κB pathway in TCGA and CGGA datasets. **b**, **c**, **e**, **f**, **h**, **i** The protein expression of the downstream genes of the NF-κB pathway after PLOD1 knockout or overexpression as measured by western blotting and gray quantitative analysis. **d**, **g**, **j**, **k** Luciferase reporter assays showed that knockout of PLOD1 in MES02-GSC and MES06-GSC could reduce the luciferase activities, while PLOD1 overexpression in PN03-GSC and PN04-GSC will correspondingly upregulate the luciferase activities. **l** CoIP was performed to find that PLOD1 can bind to IκBα. All data are shown as the mean ± SD (three independent experiments). *P < 0.05; **P < 0.01; ***P < 0.001.

To further explore the possible signaling pathways in which PLOD1 regulates the proliferation, invasion, and MES transition of GSCs, we performed GSEA on TCGA and CGGA datasets. The results showed a significant NF-κB signaling pathway enrichment in the higher PLOD1 expression group (Fig. 3a). Therefore, we examined changes in the NF-κB signaling pathway after PLOD1 regulation through western blotting. The results showed the expression levels of p-P65, p-IκBα, and p-IKKα/β were significantly downregulated after PLOD1 knockout (Fig. 3b, e, f). In contrast, the opposite results were again obtained in the PLOD1 overexpression cell lines (Fig. 3c, f, i). Then we performed a luciferase reporter gene assays, and a luciferase plasmid with the top 2000 nt of the promoter domain of P65 (pGL3-wt) and a luciferase plasmid with mutant sequences of the promoter domain (pGL3-mt) were generated. Luciferase reporter assays demonstrated that PLOD1 knockout decreased the luciferase activity of pGL3-wt in MES02-GSC and MES06-GSC, but not in pGL3-mt (Fig. 3d, g). The opposite results were obtained after PLOD1 overexpression (Fig. 3j, k). Moreover, we investigated whether there was a direct association between PLOD1 and any regulatory molecules of the NF-κB signaling. CoIP was performed and found that PLOD1 can bind to IκBα in the two CoIP assays (Fig. 3l). The above evidence proves that PLOD1 can modulate the NF-κB signaling pathway in GSCs.

### NF-κB inhibitor abrogates PLOD1-induced MES GSC-enriched tumor sphere growth and invasion in vitro

**Fig. 4:**
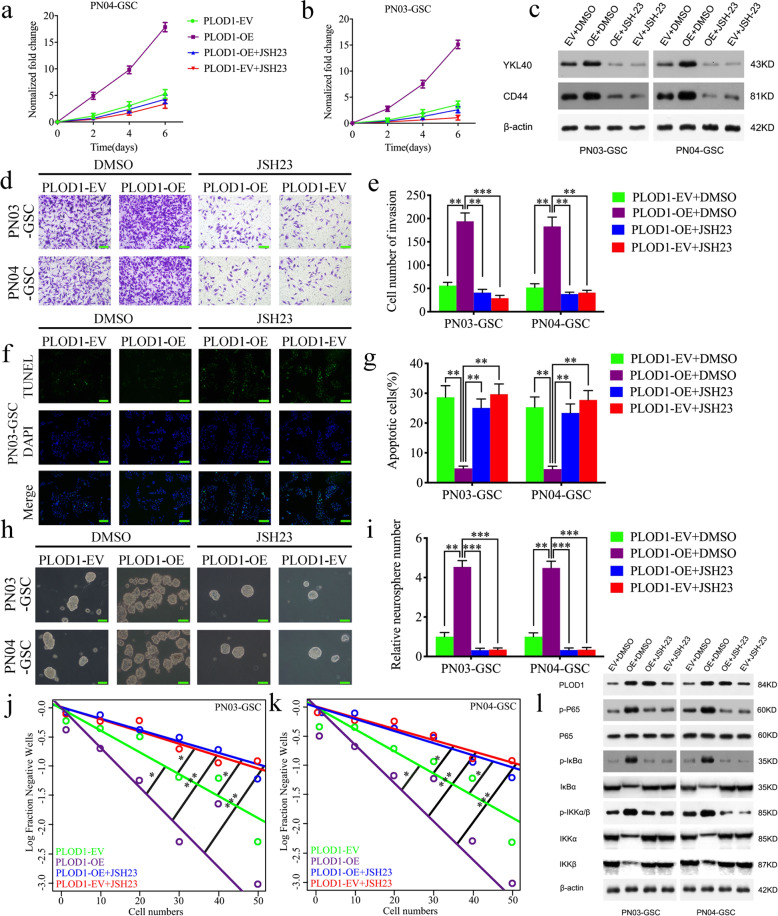
NF-κB antagonist JSH-23 abolishes the promotion of PLOD1 on malignant behavior in GBM. **a**, **b** As measured by MTS assays, JSH-23 treatment will eliminate the promotion effect of PLOD1 overexpression in PN03-GSC and PN04-GSC. **c**, **l** JSH-23 treatment regulated the protein expression of YKL40, CD44, and NF-κB downstream genes in PLOD1-overexpressed PN03-GSC and PN04-GSC as measured by western blotting. **d**, **e** Transwell assays found that the invasiveness was decreased in PLOD1 overexpression PN03-GSC and PN04-GSC after JSH-23 treatment. **f**, **g** TUNEL assays confirmed that the decreased apoptosis due to PLOD1 overexpression in PN03-GSC and PN04-GSC was significantly increased after t JSH-23 treatment. **h**–**k** JSH-23 treatment reduced the self-renewal capacity of PLOD1 overexpression PN03-GSC and PN04-GSC as measured by neurospheres formation assays and extreme limit dilution assays. Scale bar = 20 μm. All data are shown as the mean ± SD (three independent experiments). *P < 0.05; **P < 0.01; ***P < 0.001.

To further verify whether PLOD1 promotes malignant progression in GSCs through the NF-κB signaling pathway, we treated PN03-GSC, PN04-GSC cell lines after PLOD1 overexpression with JSH-23, an inhibitor of the NF-κB signaling pathway, and the blocking effect of NF-κB signaling pathway was confirmed by western blotting as lower expression levels of downstream molecules (Fig. 4l). MTS assays demonstrated that PLOD1 overexpression promoted the proliferation of PN03-GSC and PN04-GSC, but the promoting effects were reversed after administration of the JSH-23 (Fig. 4a, b). Transwell assays found that PLOD1 overexpression promoted tumor invasion, but the invasiveness of PN03-GSC and PN04-GSC was reversed after administration of the JSH-23 (Fig. 4d, e). TUNEL assays confirmed that PLOD1 overexpression inhibited apoptosis of PN03-GSC and PN04-GSC, while JSH-23 treatment obviously increased their apoptosis (Fig. 4f, g). Neurosphere formation assays also found that PLOD1 overexpression cell lines induced the production of greater relative number neurospheres, but after the inhibitor treatment, the induced neurospheres returned to the level when PLOD1 was not overexpression (Fig. 4h, i). Extreme limiting dilution assays showed a similar trend (Fig. 4j, k and Supplementary Table 1). The western blotting assays also found that PLOD1 overexpression promoted the expression of the mesenchymal marker genes CD44 and YKL40, while their expressions were downregulated after inhibitor treatment (Fig. 4c). The above evidence confirms that PLOD1 promotes the proliferation and MES transition of GSCs and inhibits their apoptosis through the NF-κB signaling pathway.

### HIF1 can directly induce the expression of PLOD1 under hypoxia

**Fig. 5:**
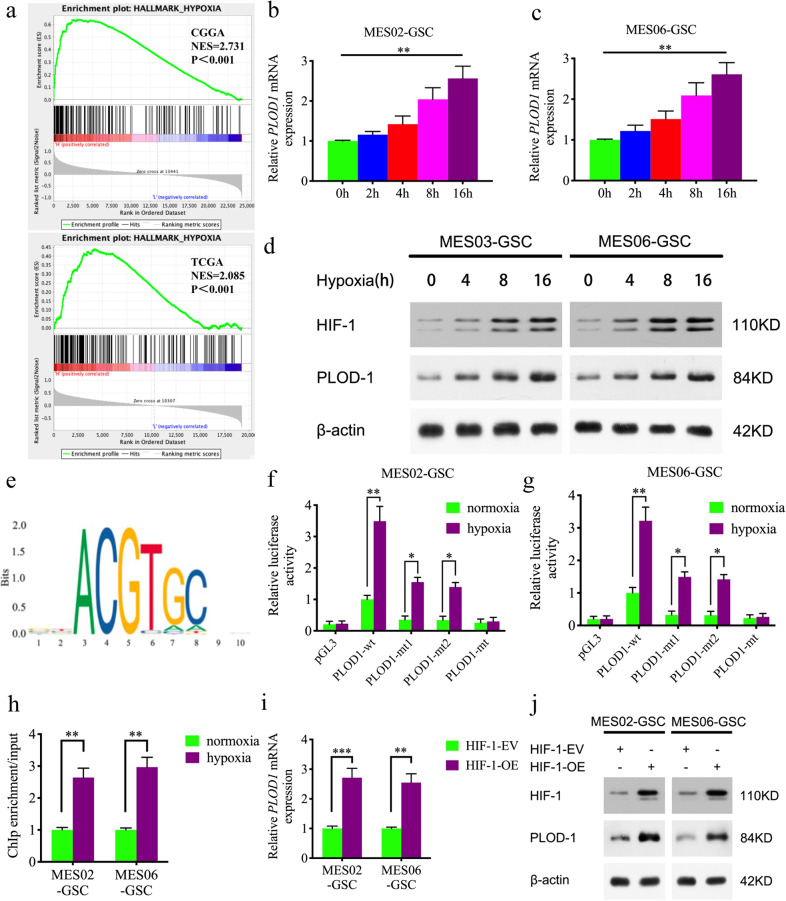
HIF-1 can transcriptionally regulate PLOD1 expression under hypoxia. **a** GSEA analysis showed obvious hypoxia enrichment when PLOD1 is highly expressed in TCGA and CGGA datasets. **b**–**d** PLOD1 expression gradually increased during the prolonged treatment under hypoxia in MES02-GSC and MES06-GSC as measured by qPCR and western blotting. **e** Sequence motif representing the shared HIF-1 binding motif (JASPAR database). **f**, **g** Luciferase reporter assays showed that hypoxia could upregulate the luciferase activities of PLOD1 in MES02-GSC and MES06-GSC. **h** ChIP-qPCR showed that HIF-1 binds to the promoter of PLOD1 under hypoxic conditions. **i**, **j** HIF-1 can upregulate the expression of PLOD1 as measured by qPCR and western blotting. All data are shown as the mean ± SD (three independent experiments). *P < 0.05; **P < 0.01; ***P < 0.001.

We further investigated the upstream mechanism of PLOD1 overexpression in GSCs. Both GSEA analyses based on the TCGA and CGGA datasets revealed significant hypoxia enrichment at a higher PLOD1 expression group (Fig. 5a). Hypoxia-inducible factor-1 (HIF-1) is the most important transcription factor under hypoxic conditions. Therefore, we further discussed whether there is a transcriptional regulation relationship between HIF-1 and PLOD1. We first treated MES02-GSC and MES06-GSC under hypoxic conditions and tested PLOD1 expression by qPCR and western blotting, demonstrating that both HIF-1 and PLOD1 expression were greatly elevated with prolonged hypoxic time (Fig. 5b–d). We further identified two possible binding sites of HIF-1 on the promoter region of PLOD1 by JASPAR analysis (Fig. 5e and Supplementary Fig. S2g). We performed the luciferase reporter gene assays and found that PLOD1-wt transfected MES02-GSC and MES06-GSC had significantly enhanced luciferase activity under hypoxia (Fig. 5f, g). Chromatin immunoprecipitation (ChIP) assays revealed PLOD1 enrichment in MES02-GSC and MES06-GSC after anti-HIF-1 treatment (Fig. 5h). In addition, qPCR and western blotting further confirmed that PLOD1 expression was remarkably upregulated after HIF-1 overexpression in MES02-GSC and MES06-GSC (Fig. 5i, j). We also detect whether HIF-2 can regulate the expression of PLOD1. However, the results showed that HIF-2 overexpression did not affect the expression of PLOD1 as measured by qPCR and western blotting (Supplementary Fig. S4). In summary, we conclude that HIF-1 directly transcriptionally regulates PLOD1 expression in GSCs under hypoxic conditions.

### PLOD1 knockout can abrogate hypoxia-induced MES GSC-enriched tumorsphere growth and invasion in vitro

**Fig. 6:**
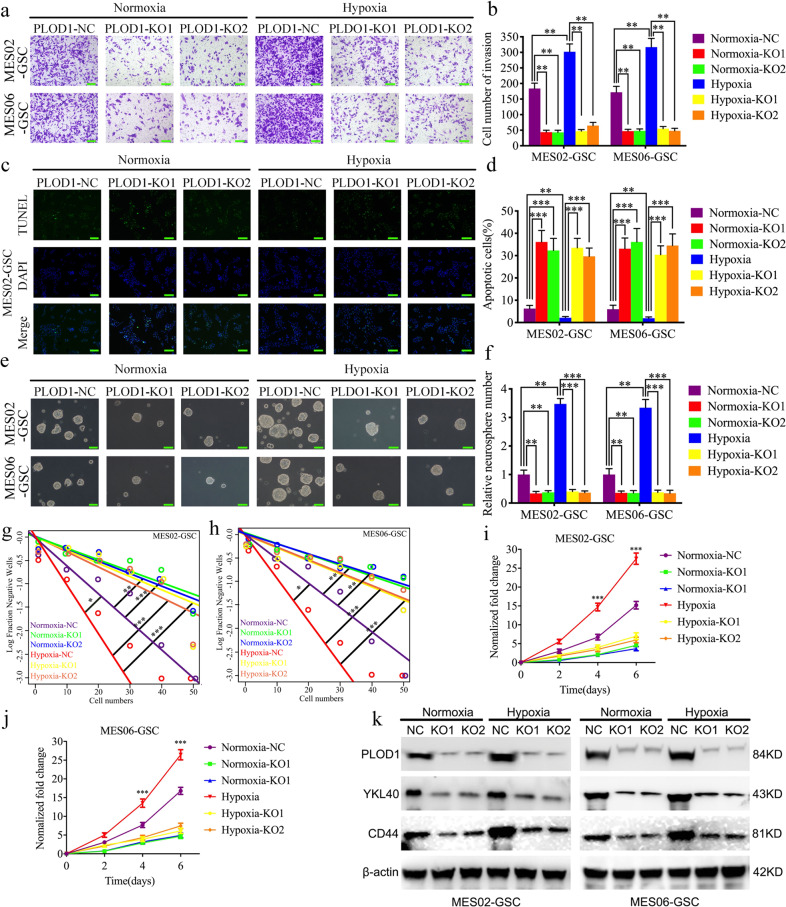
PLOD1 knockout can inhibit the malignant behaviors of GBM cells induced by hypoxia. **a**, **b** Representative transwell assays showed that hypoxia-induced invasion of GSCs was reversed after PLOD1 knockout in MES02-GSC and MES06-GSC. Scale bar = 50 μm. **c**, **d** After PLOD1 knockout in MES02-GSC and MES06-GSC, the decreased apoptosis rate induced by hypoxia was reversed, as measured by TUNEL assay. Scale bar = 50 μm. **e**–**h** In the neurosphere formation assays and extreme limit dilution assays, hypoxia’s promoting effect on neurosphere growth was reversed after PLOD1 knockout in MES02-GSC and MES06-GSC. Scale bar = 20 μm. **i**, **j** After PLOD1 knockout in MES02-GSC and MES06-GSC, hypoxia-induced cell viability was reversed, as determined by MTS assays. **k** As shown by western blotting, the protein expressions of PLOD1, YKL40, and CD44 in MES02-GSC and MES06-GSC cells upregulated under hypoxic conditions were reversed after PLOD1 knockout. All data are shown as the mean ± SD (three independent experiments). *P < 0.05; **P < 0.01; ***P < 0.001.

To further investigate the regulatory effect of PLOD1 on GSCs under hypoxic conditions, we performed MTS, transwell, TUNEL, neurosphere formation assays, and extreme limiting dilution assays on MES02-GSC after hypoxic treatment. It was found that the proliferation, invasion, and anti-apoptotic ability of tumor cells were significantly enhanced compared with the normoxic control group, which could promote the formation of neurospheres, increase the tumor formation rates, and upregulate CD44 and YKL40 expression. Subsequently, MES02-GSC and MES06-GSC with PLOD1 knockout were subjected to hypoxia treatment, and those above experiments were performed again. The MTS assays showed that the upregulated absorbance values under hypoxic conditions were reduced after PLOD1 knockout (Fig. 6i, j). Transwell assays revealed that hypoxia’s promotion of tumor invasion ability disappeared (Fig. 6a, b). TUNEL assays showed that GSCs were no longer resistant to apoptosis due to hypoxia (Fig. 6c, d). Neurosphere formation assays confirmed that the hypoxic environment’s pro-neurosphere formation effect disappeared after PLOD1 knockout (Fig. 6e, f). Similar results were obtained in extreme limiting dilution assays (Fig. 6g, h and Supplementary Table 1). Finally, we found that the expression of CD44 and YKL40 was considerably lower after PLOD1 knockout than that of both in the hypoxic state as measured by western blotting (Fig. 6k). The above results demonstrate that the hypoxic environment promotes proliferation, anti-apoptosis, and MES transition of GSCs via transcriptionally regulating PLOD1 expression.

### Hypoxia-induced PLOD1 regulates collagen I expression

Since the PLOD family can catalyze lysyl hydroxylation and directly promote collagen cross-linking and deposition [29–31], we further detect whether PLOD1 can regulate collagen expression. We first analyzed the correlation between the expression levels of PLOD1 and some collagen-related molecules in the TCGA dataset. We found significant positive correlations between PLOD1 expression and COL1A1, COL3A1, and COL4A1, while there is no correlation with COL2A1 (Supplementary Fig. S5a–d). Furthermore, it has been reported that PLOD1 can regulate collagen I’s expression rather than collagen III. According to qPCR and western blotting, our results also showed that hypoxia and PLOD1 overexpression could upregulate collagen I expression in GSCs, while PLOD1 knockout downregulated collagen I (Supplementary Fig. S5e–j).

### PLOD1 affects the tumorigenicity and MES differentiation of GSCs in vivo

**Fig. 7:**
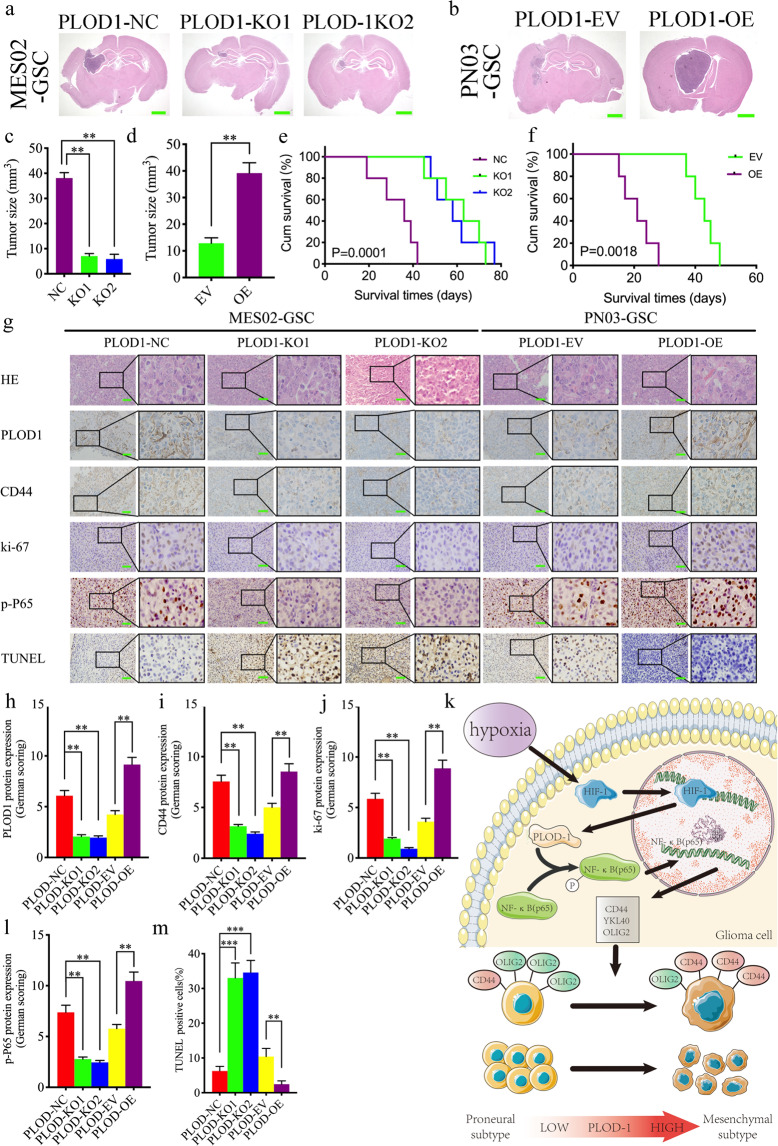
PLOD1 regulates GSC growth in vivo. **a**, **c** H&E-stained brain sections of mice with PLOD1 knockout MES02-GSC were transplanted intracranially. The brain was harvested on the 15th day after transplantation. PLOD1 knockout significantly inhibits tumor growth in vivo. Scale bar = 1 mm. **b**, **d** H&E-stained brain sections of mice with PLOD1 overexpression PN03-GSC were transplanted intracranially. The brain was harvested on the 15th day after transplantation. PLOD1 overexpression significantly enhances tumor growth in vivo. Scale bar = mm. **e** Kaplan–Meier survival curve of PLOD1 knockout MES02-GSC mice. **f** Kaplan–Meier survival curve of PLOD1 overexpression PN03-GSC mice. **g**, **m** Representative immunohistochemical staining showing the changes in PLOD1, CD44, ki-67, p-P65, and TUNEL staining in PLOD1 overexpression and knockout orthotopic xenograft models. Scale bar = 50 μm. **h**–**l** Quantitative analysis of IHC assessed according to the German immunohistochemical scoring system. **k** A working model of mesenchymal transition mediated by HIF-1/ PLOD1/ NF-κB pathway in GBM. All data are shown as the mean ± SD (three independent experiments). *P < 0.05; **P < 0.01; ***P < 0.001.

Finally, to determine whether PLOD1 can regulate tumorigenesis and MES transition of GSCs in vivo, we injected PLOD1 knockout, overexpression, and control GSCs in situ into the brains of nude mice. We found that PLOD1 knockout significantly inhibited intracranial tumor growth in MES02-GSC compared with controls (Fig. 7a, c) and prolonged survival time (mean survival: 32.8 ± 8.33, 61.2 ± 10.21, and 59.2 ± 10.19 days; Fig. 7e). In contrast, PLOD1 overexpression cell line PN03-GSC had a significantly larger tumor volume (Fig. 7b, d) and a notable shorter median survival time than the empty vector control (mean survival: 42.6 ± 3.83 and 21 ± 4.69 days; Fig. 7f). We further performed H&E staining and immunohistochemical staining of tumorigenic tissues, and the intensity and expression levels of PLOD1, CD44, ki-67, and p-P65 were clearly lower in the PLOD1 knockout group than in the control group. In contrast, the opposite results were obtained after PLOD1 overexpression (Fig. 7g). Moreover, We supplied quantitative analysis of immunohistochemistry (IHC) assessed according to the German immunohistochemical scoring system [32] (Fig. 7h, i, j, l). Furthermore, we found that the TUNEL-positive rate increased noticeably after PLOD1 knockout, whereas PLOD1 overexpression decreased the rate significantly (Fig. 7g, m). The above results show that PLOD1 can promote tumorigenesis, MES transition, and enhance malignant behaviors in nude mice. In conclusion, we demonstrate the possible mechanism by which PLOD1 regulates the behavior of GSCs in terms of genesis, proliferation, anti-apoptosis, and MES transition through a model diagram (Fig. 7k).

## Discussion

Surgical resection combined with radiotherapy and chemotherapy is the classical treatment for GBM, but it is often susceptible to recurrence and ultimately leads to patient deaths [33, 34]. Despite the emergence of new therapies such as immunotherapy, stem cell therapy, and nanotechnology in recent years, the GBM treatment prognosis remains poor [35]. Molecularly targeted therapies targeting oncogenic genes associated with GBM genesis and development are increasingly considered a promising way to cure GBM [36, 37]. MES transition is an important phenomenon of increased GBM aggressiveness, radiotherapy tolerance, and relapse [8, 12, 38, 39]. Therefore, identifying the molecular mechanisms that regulate malignant biological behaviors, especially MES transition, is expected to improve the treatment efficacy of GBM and guide molecular targeted therapy.

Our study firstly performed bioinformatics analysis based on TCGA and CGGA datasets. PLOD1 was overexpression in mesenchymal and IDH wild-type GBM and was strongly associated with poor prognosis. Subsequently, PLOD1 expression in clinical specimens was also confirmed to be highest in the mesenchymal GBM and lowest in the proneural subtype with the lowest degree of malignancy. Moreover, Kaplan–Meier survival analysis showed that in mesenchymal GBM, patients with high PLOD1 expression had shorter median survival times than patients with low PLOD1 expression. The proneural and mesenchymal subtypes are more clinically consistent in their respective subtypes [40], with the proneural subtype GBM exhibiting a higher proportion of IDH1 mutations and showing a favorable prognosis. In contrast, the mesenchymal subtype GBM reflects more IDH1 wild type and is associated with a poor prognosis [5, 41]. Therefore, PLOD1 may be a novel oncogene for glioma occurrence, progression, and MES transition in mesenchymal subtype GBM. Besides, PLOD1 is also significantly overexpression in a wide range of malignancies, including esophageal squamous cell carcinoma, gastric cancer, and colorectal cancer, and is significantly associated with poor patient outcomes [21, 22].

The PLOD family catalyzes lysyl hydroxylation and directly promotes collagen cross-linking and deposition, leading to carcinogenesis and regulating proliferation, migration, invasion, apoptosis, and several cancer cells [29–31]. In esophageal squamous cell carcinoma, the expression of tumor suppressor gene esophageal cancer-related gene 4 was negatively correlated with PLOD1 and PLOD2 [21]. However, the function of PLOD1 in GBM has not been reported. Our study demonstrated the oncogenic potential of PLOD1 by modulating PLOD1 expression in patient-derived GSCs, demonstrating that PLOD1 overexpression promotes GSCs proliferation, invasion, anti-apoptosis, and MES transition in vitro and vivo, whereas PLOD1 knockout has the opposite effects. Acetyl heparan sulfate proteoglycan is a key component of the brain ECM and obviously increased in glioma [42], acting as a reservoir for heparin-binding angiogenic growth factors such as fibroblast growth factor and vascular endothelial growth factor [43], regulating tumor cell niche, angiogenesis, and invasion mechanisms [44]. Moreover, our study has confirmed that PLOD1 can upregulate collagen I expression, which suggests that PLOD1 may regulate collagen cross-linking and deposition, participate in ECM, and further affect the course of GBM disease.

We then explored the possible downstream mechanisms of tumor-promoting effects of PLOD1, and GSEA analysis revealed enrichment of the NF-κB signaling pathway at higher PLOD1 expression. The main NF-κB dimer in GBM cells consists of p50 and p65, and p65 is generally retained in the cytoplasm at rest [45, 46]. Stimulation from cytokines and oncogenes can activate IKK, lead to the nuclear translocation of p65, and function as a transcriptional factor in cancer cells. Given the critical role of p65 in NF-κB signaling, most GBM studying of NF-κB focused on this subunit [24]. We conducted a series of experiments to find that overexpression of PLOD1 in GSCs activates the NF-κB signaling pathway, leading to phosphorylation and nuclear translocation of the P65 subunit, thereby modulating the transcriptional activity of molecules downstream of the NF-κB signaling pathway. Moreover, since PLOD1 can directly bind to IκBα according to the CoIP, it suggested that PLOD1 may activate the NF-κB signaling pathway through binding IκBα to induce phosphorylation of IκBα, but this requires further research of validation. Previously, it has been shown that proneural GBM can be transformed into a mesenchymal subtype via a TNF-α/NF-κB-dependent manner, with CD44 subpopulation enrichment and radiotherapy tolerance [12]. In addition, invasive tumor-associated macrophages and microglia promote the MES transition of GBM by releasing cytokines into the microenvironment that activating NF-κB through P65 phosphorylation [12, 47–49]. We further performed rescue assays and found that NF-kB inhibitors treatment effectively inhibited glioma proliferation, invasion, migration, MES transition, and other malignant behaviors [50], resulting in the disappearance of all promoting effects caused by PLOD1 overexpression. Thus, PLOD1 plays an important role in GSCs MES transition and malignant behaviors through NF-κB signaling.

We further investigated the upstream mechanism of PLOD1 overexpression in GBM. GSEA analysis was performed on the TCGA and CGGA datasets and find that there was a marked hypoxic enrichment in higher expression of PLOD1. Moreover, it has been reported that in breast cancer, HIF-1 activates PLOD1 and PLOD2 transcription under hypoxic conditions, regulates collagen cross-linking, and promotes lymph node and lung metastasis [20, 26]. At the same time, hypoxia is also an important feature of GBM cells, with effects such as promoting glioma cells growth, invasion, and MES transition [27, 51]. According to the experiments on patient-derived GSCs, HIF-1 can strongly promote PLOD1 transcription and expression under hypoxic environment. Hypoxia can also promote GSCs proliferation, invasion, resistance to apoptosis, and MES transition. However, hypoxia’s tumor-promoting effects were reversed after PLOD1 knockout, suggesting that PLOD1 is an important downstream gene of the tumor-promoting effects under hypoxic environment on GSCs. Thus, hypoxia due to rapid glioma growth can activate HIF-1 [52], promoting the proliferation, invasion, and anti-apoptosis of GSCs through transcriptional regulation of PLOD1, leading to a series of malignant behaviors [53, 54]. Also, it may be possible that PLOD1, together with HIF-1, constitutes a positive loop of malignancy in GSCs, which in turn induces MES transition and radiotherapy tolerance in GBM.

In conclusion, we found that PLOD1 is closely associated with the occurrence, development, and poor prognosis of GBM, especially in mesenchymal GBM, and that PLOD1 regulates GSCs proliferation, invasion, MES transition, and other malignant behaviors through the NF-κB signaling pathway. Moreover, overexpression of PLOD1 was transcriptionally regulated by HIF-1 under a hypoxic environment as its upstream mechanism. PLOD1 is likely to be an effective therapeutic target for GBM, especially mesenchymal GBM and provide a possible treatment target for GBM therapy.

## Materials and methods

### Ethics

This study was approved by the institutional review board of the Shanghai General Hospital. In this study, patient samples were taken from 93 patients with GBM collected from January 2006 to December 2011 at the First Affiliated Hospital of China Medical University (Shenyang, China), and informed consent was refined. The expression of mRNA markers (EGFR, FN1, YKL40, NEFL, PDGFRA, and OLIG2) was examined using real-time PCR for the molecular classification [55]. All animal experiments were conducted under the supervision of the Animal Ethics Committee of Shanghai Jiao Tong University School of Medicine. Clinical information for these samples is outlined in Supplementary Table 2.

### Cell treatment

The NHA was purchased from American Type Culture Collection (ATCC, Manassas, VA, USA). Patient-derived GSCs (PN03-GSC, PN04-GSC, PN09-GSC, MES02-GSC, MES06-GSC, and MES13-GSC) were isolated and neurosphere cultures were performed as previously reported [28]. The detailed clinicopathological information is presented in Supplementary Table 3. Briefly, freshly resected clinical glioma tissues were dissociated into single cells and cultured in Dulbecco’s modified Eagle’s medium (DMEM) with B27 (1:50), recombinant human (rh) basic fibroblast growth factor (20 ng/mL), and rh-epidermal growth factor (20 ng/mL, Gibco, Gaithersburg, MD, USA). Neurospheres were then collected and grown in neurosphere medium following standard procedures. GSCs were differentiated and cultured with 10% fetal bovine serum in DMEM. GSCs with tumor stem cell properties were assessed by self-renewal and functional assays of tumor formation in vivo. The expression of stem cell markers (CD133, CD44, and nestin+) was detected by flow cytometry or immunofluorescence, and the multi-lineage differentiation ability of GSCs was detected by immunofluorescence. CD44^high^ and CD44^low^ GSCs were isolated by magnetic cell sorting using CD44 microbeads (#130-095-194, Miltenyi Biotec, Bergisch Gladbach, Germany). To induce hypoxia, GSCs were cultured in a hypoxia chamber with 94% N_2_, 5% CO_2_, and 1% O_2_ at 37 °C. All GSCs analyzed were cultured with less than 20 generations. The cell lines and GSCs have passed mycoplasma and the short tandem repeat DNA profiling test.

### Lentiviral vector construction and transfection

CRISPR-Cas9-mediated PLOD1 knockout and PLOD1 overexpression based on lentivirus-based vector transfection was performed with technical support from Gene-Chem (Shanghai, China). Engineered two sgRNA sequences for PLOD1 knockout: PLOD1-KO1: 5′-CTTGAAGCGACGGAATCCCT-3′, PLOD1-KO2: 5′-GAGCTGAGCGCTTGAAGCGA-3′. After transfection, all cells were examined for resistance to puromycin (Sigma, Santa Clara, CA, USA) for 15 days at a concentration of 10 μg/mL. PLOD1 knockout or overexpression was validated by qPCR and western blotting.

### Real-time quantitative reverse transcription PCR (qRT-PCR)

Mini-BEST Universal RNA Extraction kit (TaKaRa, Kyoto, Japan) was used to isolate total RNA according to the manufacturer’s instructions. Prime-Script RT Master Mix (TaKaRa) was used to synthesis first-strand cDNA, followed by qPCR (PCR LightCycler480, Roche Diagnostics Ltd., Basel, Switzerland) detection using SYBR Green Master Mix (TaKaRa). Each sample was run four times, and β-actin was used as the internal control. The sequences of the PCR primer pairs were as follows: PLOD1, forward 5′-AAGCCGGAGGACAACCTTTTA-3′ and reverse 5′-GCGAAGAGAATGACCAGATCC-3′; COL1A1, forward 5′-GAGGGCCAAGACGAAGACATC-3′ and reverse 5′-CAGATCACGTCATCGCACAAC-3′; β-actin, forward 5′-CATGTAC GTTGCTATCCAGGC-3′ and reverse 5′-CTCCTTAAT GTCACGCACGAT-3′.

### Western blotting

Western blotting was performed as described previously [28]. A cell protein extraction kit (KeyGen Biotechnology, Nanjing, China) was used to isolate the cell protein from PLOD1-knockout, -overexpressing, or control cells. The primary antibodies against PLOD1 (1:1000, #SAB1301577, Sigma‐Aldrich), HIF-1 (1:500, #ab1, Abcam Technology, Cambridge, UK), YKL40 (1:1000, #ab180569, Abcam), CD44 (1:2000, #ab157107, Abcam), Olig2 (1:2000, #ab109186, Abcam), p-P65 (1:2000, #ab86299, Abcam), P65 (1:2000, #ab16502, Abcam), p-IκBα (1:10000, #ab133462, Abcam), IκBα (1:1000, #ab76429, Abcam), p-IKKα/β (1:500, #ab194528, Abcam), IKKα (1:1000, #ab109749, Abcam), IKKβ (1:500, #ab97406, Abcam), Collagen I (1:1000, #ab34710, Abcam), HIF-2 (1:1000, #ab243861, Abcam), and β-actin (1:2000, #66009-1-Ig, ProteinTech, Chicago, IL, USA) were used. The bands on each membrane were detected using an ECL kit (Beyotime Biotechnology, Beijing, China) and IMAGE J software (National Institutes of Health, Bethesda, MD, USA) was used for quantification.

For immunoprecipitation, RIPA lysis buffer containing proteasome inhibitor (Beyotime Biotechnology) was used to lyse the cell pellets. The total cell lysates were immunoprecipitated at 4 °C for 12 h using a rabbit monoclonal antibody against PLOD1 or a mouse monoclonal antibody against IκBα (Abcam) that was prebound to protein A-Sepharose 4B beads (GE Healthcare, Pittsburgh, PA, USA). Appropriate isotype antibodies were used as controls (Abcam). The beads were washed and boiled in SDS buffer, and the eluates were analyzed by western blotting.

### Cell viability assays

According to the manufacturer’s instructions, cell viability was measured using a CellTiter 96^®^ AQueous Non-Radioactive cell proliferation assay kit (Promega, Madison, WI, USA). Briefly, cells were cultured in 96-well plates at a density of 1 × 10^3^ cells/well for 24, 48, 72, 96, and 120 h, respectively. Then 20 μL of MTS was added into each well, followed by 3 h incubation at 37 °C. An ultraviolet spectrophotometer (Thermo Fisher Scientific, Waltham, MA, USA) was used to detect the absorbance at 495 nm.

### Transwell assay

The transwell assay was performed as previously described [28]. PLOD1-knockout, -overexpressing, or control cells were treated with FBS-free DMEM and applied to matrigel-coated filters. After 20 h, the cells that had invaded the lower compartment were counted and photographed under a microscope.

### TUNEL assay

TUNEL assay was performed to detect the apoptotic cells after 3 days in culture using the TdT-FragEL DNA Fragmentation Detection Kit (QIA33, Merck, Darmstadt, Germany) according to the manufacturer’s instructions. Apoptosis of tissue samples was evaluated using another TUNEL apoptosis detection kit (DAB type, Wanlei Biotechnology). TUNEL-positive cells were counted, and the apoptotic rate was calculated as follows: positive cells / (positive cells + negative cells) × 100%.

### Immunofluorescence

Immunofluorescence was performed as described previously [56]. The cells were fixed with 4% paraformaldehyde, permeabilized with 0.5% Triton X-100, blocked with 5% BSA, and incubated with primary antibody overnight at 4 °C, followed by FITC- or rhodamine-conjugated secondary antibody. The primary antibodies against PLOD1 (Sigma‐Aldrich), CD133 (1:1000, #ab222782, Abcam), CD44 (Abcam), nestin+ (1:500, #ab18102, Abcam), GFAP (1:1000, #ab4674, Abcam), and β III tubulin (1:1000, #ab7751, Abcam) were the same as described above. DAPI (Sigma‐Aldrich) was used to stain the nuclei. A laser scanning confocal microscope was used to detect and photograph immunofluorescence expression (Olympus, Tokyo, Japan).

### Neurosphere formation assays

The neurosphere formation assay was performed as previously described [57]. Briefly, GSCs were seeded into 24-well plates at a density of 200 cells/well and incubated in fresh medium for 7 days. The relative number of neurospheres were counted using an optical microscope (Olympus). In vitro extreme limiting dilution method, GSCs were seeded at a gradient of 1, 10, 20, 30, 40, or 50 cells per well to 96 in well plates, 10 replicates per gradient. 7 days later, the number of neurospheres in each well was observed, and the spheres were calculated using extreme limit dilution analysis creating efficiency [58].

### Immunohistochemistry (IHC)

IHC was performed as described previously [56]. Briefly, tissue samples were paraffin-embedded, cut into 4-μm sections, and examined by using a combination of anti-PLOD1 (Sigma‐Aldrich), CD44, YKL40, Ki-67, and p-P65 (Abcam) for primary antibody labeling. Sections were imaged with an optical microscope (Olympus), and staining intensity was assessed according to the German immunohistochemical scoring system [32].

### Luciferase activity analysis

Luciferase reporter assays were performed as previously described [28]. Briefly, NF-κB and PLOD1 reporter plasmids were constructed by Gene-Chem (Shanghai, China). Cells were seeded into 96-well plates at a density of 5 × 10^3^ cells per well and transfected with different plasmids. After 48 h, the cells were lysed, and luciferase activity was measured using the Dual-Luciferase Reporter Assay System (Promega), according to the manufacturer’s instructions. Each experiment was independently repeated three times.

### Chromatin immunoprecipitation (ChIP) assays

ChIP assays were performed using the EZ-ChIP™ Immunoprecipitation Kit (Millipore, Billerica, MA, USA) according to the manufacturer’s instructions. The chromatin complexes were immunoprecipitated with anti-HIF1 antibody (Abcam), and the purified DNA samples were analyzed with qPCR using primer pairs for the HIF-1-binding site in the PLOD1 promoter f: 5′- ATCCGTGCCCACCCTCA-3′ and r: 5′- AGAAAGAGGGCGGTGAGA-3′. All reactions were performed in triplicate.

### Xenograft experiments

The animal experiments were approved by the Guidelines for the Care and Use of Laboratory Animals and the Medical Ethics Committee of Shanghai General Hospital (Shanghai, China). Six-week-old female BALB/c nude mice were obtained from Beijing Vital River Laboratory Animal Technology and bred in laminar flow cabinets under specific pathogen-free conditions. Transfected GSCs (1 × 10^5^ cells) were injected into the mouse brain at 2 mm lateral and 2 mm anterior to the bregma using a stereotaxic apparatus. The mice were randomly divided into five groups and five mice in each group were observed daily for neurological symptoms or death, and tumor growth was evaluated. The tumor volume was calculated according to the formula: *V* = (*D* × *d* [2]) / 2, where *D* represents the longest diameter and *d* represents the shortest diameter. The accessing process was conducted by an assessor blind to treatment allocation.

### Bioinformatic analyses

The data on mRNA expression and clinical samples from patients with GBM were obtained from The Cancer Genome Atlas (TCGA, http://cancergenome.nih.gov) in the HG-U133A platform and the Chinese Glioma Genome Atlas (CGGA, http://www.cgga.org.cn) in RNA-seq platform. Gene-set enrichment analysis (GSEA, http://www.broadinstitute.org/gsea/index.jsp) was used to detect any possible signal pathway sets of genes showing statistically significant differences between higher and lower PLOD1 expression groups. The functional relationships between PLOD1 and other genes were tested by two-sided Pearson’s product-moment correlation. GSEA was also used to detect sets of genes from signaling pathways that showed statistically significant differences between higher and lower PLOD1 expression groups.

### Statistical analysis

Statistical analysis was performed using SPSS 22.0 software (IBM, Armonk, N.Y, USA). All the data meet the assumption of normal distribution. All experiments were repeated at least three times, and the results are presented as the mean ± standard deviation. The chi-square test and t-test were used to evaluate the statistical significance between groups. Differences in survival rates were analyzed using the log-rank test and Kaplan–Meier analysis. All statistical tests were two-sided, and statistical significance was defined as a *p*-value < 0.05.

## Supplementary information

Supplementary figure legend

Supplementary Table1

Supplementary Table2

Supplementary Table3

Supplementary Figure 1

Supplementary Figure 2

Supplementary Figure 3

Supplementary Figure 4

Supplementary Figure 5
