# Supplementary material for: Hypoxia-induced PLOD1 overexpression contributes to the malignant phenotype of glioblastoma via NF-κB signaling
Source: Oncogene. 2021 Jan 8;40(8):1458–75. doi: 10.1038/s41388-020-01635-y (PMC7906902; doi:10.1038/s41388-020-01635-y)
Supplement: Supplementary file 1 — Supplementary figure legend [file 41388_2020_1635_MOESM1_ESM.docx]

Supplementary figure 1

PLOD1 is expressed at a higher level in the mesenchymal GBM and is associated with poor patient survival in the TCGA dataset.

a, b, c, d: The mRNA expression of PLOD1 is shown according to WHO grades, GBM or LGG, IDH status and the molecular subtypes in the TCGA datasets.

e, f: GSEA analysis showed that mesenchymal subtype enrichment occurs when PLOD1 is highly expressed, while proneural subtype is enriched when PLOD1 is lowly expressed in the TCGA dataset.

g: The expression of PLOD1 is positively correlated with the expression of mesenchymal related genes, which is opposite to the proneural related genes in the TCGA datasets.

h: Kaplan-Meier analysis of GBM patients with high PLOD1 expression versus low PLOD1 expression in the TCGA dataset.

All data are shown as the mean ± SD (three independent experiments). *P < 0.05; **P < 0.01; ***P < 0.001.

Supplementary figure 2

Isolation and validation of patient-derived glioma stem cells.

a: H&E stained images of the original patient tumors of MES02-GSC, PN03-GSC, PN04-GSC, MES06-GSC, PN09-GSC, MES13-GSC.

b: CD133 or CD44 expression in GSCs was detected by flow cytometry.

c: Neurospheres composed of CD133^+^ / nestin^+^ or CD44^+^ / nestin^+^ GSCs were isolated from the primary culture. Scale bar = 50 μm.

d: GSCs adhered and differentiated into GFAP- or β-III tubulin-positive cells. Scale bar = 50 μm.

e: PLOD1 mRNA expression in patient-derived GSCs and NHA as measured by qPCR.

f: The protein expression of PLOD1, YKL40, CD44 and OLIG2 in patient-derived GSCs as measured by western blotting.

g: A schematic diagram of the changes of base pairs during wild-type mutation of PLOD1.

h, i: Detection of PLOD1 expression in CD44-high and CD44-low GSCs by qPCR and western blotting.

All data are shown as the mean ± SD (three independent experiments). *P < 0.05; **P < 0.01; ***P < 0.001.

Supplementary figure 3

PLOD1 overexpression promotes GSCs' malignant behaviors in vitro.

a, b: The expression of PLOD1 in PN03-GSC and PN04-GSC after transfection of the PLOD1 overexpression plasmids as measured by western blotting and qPCR.

c, f: PLOD1 overexpression significantly increased the proliferation of PN03-GSC and PN04-GSC in MTS assays.

d, e: Transwell assay showed the invasion of PN03-GSC and PN04-GSC after PLOD1 overexpression. Scale bar = 50μm.

g, h: PLOD1 overexpression can significantly decrease the apoptosis of PN03-GSC and PN04-GSC as measured by TUNEL. Scale bar = 50μm.

i, n: The protein expression of YKL40 and CD44 in PLOD1 overexpression PN03-GSC and PN04-GSC as measured by western blotting and gray quantitative analysis.

j, k, l, m: Representative images of neurospheres and extreme limiting dilution assays showed tumor formation rate upregulated after PLOD1 overexpression in PN03-GSC and PN04-GSC. Scale bar = 20μm.

All data are shown as the mean ± SD (three independent experiments). *P < 0.05; **P < 0.01; ***P < 0.001.

Supplementary figure 4

HIF-2 overexpression did not affect the expression of PLOD1

a, b: HIF-1 can not affect the expression of PLOD1 as measured by qPCR and western blotting.

All data are shown as the mean ± SD (three independent experiments). *P < 0.05; **P < 0.01; ***P < 0.001.

Supplementary figure 5

Hypoxia induced PLOD1 regulates Collagen I expression

a, b, c, d: The expression of PLOD1 is positively correlated with the expression of COL1A1, COL3A1, COL4A1 without COL2A1 in TCGA database.

e, h: The expression of COL1A1 and Collagen I in MES02-GSC after transfection of PLOD1-KO1, PLOD1-KO2 or negative control as measured by western blotting and qPCR.

f, i: The expression of COL1A1 and Collagen I in PN03-GSC after transfection of the PLOD1 overexpression plasmids as measured by western blotting and qPCR.

g, j: The expression of COL1A1 and Collagen I in MES02-GSC after hypoxia treatment as measured by western blotting and qPCR.

All data are shown as the mean ± SD (three independent experiments). *P < 0.05; **P < 0.01; ***P < 0.001.
