# Supplementary material for: Hypoxia-induced PLOD1 overexpression contributes to the malignant phenotype of glioblastoma via NF-κB signaling
Source: Oncogene. 2021 Jan 8;40(8):1458–75. doi: 10.1038/s41388-020-01635-y (PMC7906902; doi:10.1038/s41388-020-01635-y)
Supplement: Supplementary file 2 — Supplementary Table1 [file 41388_2020_1635_MOESM2_ESM.docx]

**Table S1 The detailed stem cell frequencies of GSCs as measured through in vitro limiting dilution assays.**

|  | | number of cells in each culture | number of cultures tested | number of positive cultures | label for the population group to which cells belong |
| --- | --- | --- | --- | --- | --- |
| Figure3 | MES02-GSC | 1 | 10 | 4 | PLOD1-NC |
|  |  | 10 | 10 | 4 | PLOD1-NC |
|  |  | 20 | 10 | 6 | PLOD1-NC |
|  |  | 30 | 10 | 8 | PLOD1-NC |
|  |  | 40 | 10 | 8 | PLOD1-NC |
|  |  | 50 | 10 | 10 | PLOD1-NC |
|  |  | 1 | 10 | 1 | PLOD1-KO1 |
|  |  | 10 | 10 | 2 | PLOD1-KO1 |
|  |  | 20 | 10 | 3 | PLOD1-KO1 |
|  |  | 30 | 10 | 5 | PLOD1-KO1 |
|  |  | 40 | 10 | 6 | PLOD1-KO1 |
|  |  | 50 | 10 | 9 | PLOD1-KO1 |
|  |  | 1 | 10 | 2 | PLOD1-KO2 |
|  |  | 10 | 10 | 2 | PLOD1-KO2 |
|  |  | 20 | 10 | 3 | PLOD1-KO2 |
|  |  | 30 | 10 | 5 | PLOD1-KO2 |
|  |  | 40 | 10 | 5 | PLOD1-KO2 |
|  |  | 50 | 10 | 6 | PLOD1-KO2 |
|  | MES06-GSC | 1 | 10 | 3 | PLOD1-NC |
|  |  | 10 | 10 | 5 | PLOD1-NC |
|  |  | 20 | 10 | 6 | PLOD1-NC |
|  |  | 30 | 10 | 7 | PLOD1-NC |
|  |  | 40 | 10 | 9 | PLOD1-NC |
|  |  | 50 | 10 | 10 | PLOD1-NC |
|  |  | 1 | 10 | 2 | PLOD1-KO1 |
|  |  | 10 | 10 | 2 | PLOD1-KO1 |
|  |  | 20 | 10 | 3 | PLOD1-KO1 |
|  |  | 30 | 10 | 4 | PLOD1-KO1 |
|  |  | 40 | 10 | 5 | PLOD1-KO1 |
|  |  | 50 | 10 | 8 | PLOD1-KO1 |
|  |  | 1 | 10 | 2 | PLOD1-KO2 |
|  |  | 10 | 10 | 3 | PLOD1-KO2 |
|  |  | 20 | 10 | 4 | PLOD1-KO2 |
|  |  | 30 | 10 | 5 | PLOD1-KO2 |
|  |  | 40 | 10 | 6 | PLOD1-KO2 |
|  |  | 50 | 10 | 7 | PLOD1-KO2 |
| Figure 5 | PN03-GSC | 1 | 10 | 2 | PLOD1-EV |
|  |  | 10 | 10 | 3 | PLOD1-EV |
|  |  | 20 | 10 | 4 | PLOD1-EV |
|  |  | 30 | 10 | 7 | PLOD1-EV |
|  |  | 40 | 10 | 7 | PLOD1-EV |
|  |  | 50 | 10 | 9 | PLOD1-EV |
|  |  | 1 | 10 | 3 | PLOD1-OE |
|  |  | 10 | 10 | 5 | PLOD1-OE |
|  |  | 20 | 10 | 7 | PLOD1-OE |
|  |  | 30 | 10 | 9 | PLOD1-OE |
|  |  | 40 | 10 | 8 | PLOD1-OE |
|  |  | 50 | 10 | 10 | PLOD1-OE |
|  |  | 1 | 10 | 1 | PLOD1-OE+JSH23 |
|  |  | 10 | 10 | 2 | PLOD1-OE+JSH23 |
|  |  | 20 | 10 | 3 | PLOD1-OE+JSH23 |
|  |  | 30 | 10 | 4 | PLOD1-OE+JSH23 |
|  |  | 40 | 10 | 5 | PLOD1-OE+JSH23 |
|  |  | 50 | 10 | 7 | PLOD1-OE+JSH23 |
|  |  | 1 | 10 | 1 | PLOD1-EV+JSH23 |
|  |  | 10 | 10 | 1 | PLOD1-EV+JSH23 |
|  |  | 20 | 10 | 2 | PLOD1-EV+JSH23 |
|  |  | 30 | 10 | 5 | PLOD1-EV+JSH23 |
|  |  | 40 | 10 | 6 | PLOD1-EV+JSH23 |
|  |  | 50 | 10 | 6 | PLOD1-EV+JSH23 |
|  | PN04-GSC | 1 | 10 | 3 | PLOD1-EV |
|  |  | 10 | 10 | 3 | PLOD1-EV |
|  |  | 20 | 10 | 5 | PLOD1-EV |
|  |  | 30 | 10 | 6 | PLOD1-EV |
|  |  | 40 | 10 | 7 | PLOD1-EV |
|  |  | 50 | 10 | 9 | PLOD1-EV |
|  |  | 1 | 10 | 4 | PLOD1-OE |
|  |  | 10 | 10 | 5 | PLOD1-OE |
|  |  | 20 | 10 | 7 | PLOD1-OE |
|  |  | 30 | 10 | 7 | PLOD1-OE |
|  |  | 40 | 10 | 9 | PLOD1-OE |
|  |  | 50 | 10 | 10 | PLOD1-OE |
|  |  | 1 | 10 | 1 | PLOD1-OE+JSH23 |
|  |  | 10 | 10 | 1 | PLOD1-OE+JSH23 |
|  |  | 20 | 10 | 3 | PLOD1-OE+JSH23 |
|  |  | 30 | 10 | 4 | PLOD1-OE+JSH23 |
|  |  | 40 | 10 | 6 | PLOD1-OE+JSH23 |
|  |  | 50 | 10 | 7 | PLOD1-OE+JSH23 |
|  |  | 1 | 10 | 1 | PLOD1-EV+JSH23 |
|  |  | 10 | 10 | 2 | PLOD1-EV+JSH23 |
|  |  | 20 | 10 | 2 | PLOD1-EV+JSH23 |
|  |  | 30 | 10 | 4 | PLOD1-EV+JSH23 |
|  |  | 40 | 10 | 6 | PLOD1-EV+JSH23 |
|  |  | 50 | 10 | 6 | PLOD1-EV+JSH23 |
| Figure 6 | MES02-GSC | 1 | 10 | 3 | Normoxia-NC |
|  |  | 10 | 10 | 5 | Normoxia-NC |
|  |  | 20 | 10 | 6 | Normoxia-NC |
|  |  | 30 | 10 | 7 | Normoxia-NC |
|  |  | 40 | 10 | 9 | Normoxia-NC |
|  |  | 50 | 10 | 10 | Normoxia-NC |
|  |  | 1 | 10 | 1 | Normoxia-KO1 |
|  |  | 10 | 10 | 3 | Normoxia-KO1 |
|  |  | 20 | 10 | 4 | Normoxia-KO1 |
|  |  | 30 | 10 | 4 | Normoxia-KO1 |
|  |  | 40 | 10 | 5 | Normoxia-KO1 |
|  |  | 50 | 10 | 8 | Normoxia-KO1 |
|  |  | 1 | 10 | 2 | Normoxia-KO2 |
|  |  | 10 | 10 | 2 | Normoxia-KO2 |
|  |  | 20 | 10 | 3 | Normoxia-KO2 |
|  |  | 30 | 10 | 5 | Normoxia-KO2 |
|  |  | 40 | 10 | 6 | Normoxia-KO2 |
|  |  | 50 | 10 | 8 | Normoxia-KO2 |
|  |  | 1 | 10 | 4 | Hypoxia-NC |
|  |  | 10 | 10 | 6 | Hypoxia-NC |
|  |  | 20 | 10 | 8 | Hypoxia-NC |
|  |  | 30 | 10 | 9 | Hypoxia-NC |
|  |  | 40 | 10 | 10 | Hypoxia-NC |
|  |  | 50 | 10 | 10 | Hypoxia-NC |
|  |  | 1 | 10 | 2 | Hypoxia-KO1 |
|  |  | 10 | 10 | 2 | Hypoxia-KO1 |
|  |  | 20 | 10 | 3 | Hypoxia-KO1 |
|  |  | 30 | 10 | 5 | Hypoxia-KO1 |
|  |  | 40 | 10 | 6 | Hypoxia-KO1 |
|  |  | 50 | 10 | 9 | Hypoxia-KO1 |
|  |  | 1 | 10 | 3 | Hypoxia-KO2 |
|  |  | 10 | 10 | 3 | Hypoxia-KO2 |
|  |  | 20 | 10 | 4 | Hypoxia-KO2 |
|  |  | 30 | 10 | 5 | Hypoxia-KO2 |
|  |  | 40 | 10 | 6 | Hypoxia-KO2 |
|  |  | 50 | 10 | 9 | Hypoxia-KO2 |
|  | MES06-GSC | 1 | 10 | 2 | Normoxia-NC |
|  |  | 10 | 10 | 4 | Normoxia-NC |
|  |  | 20 | 10 | 5 | Normoxia-NC |
|  |  | 30 | 10 | 7 | Normoxia-NC |
|  |  | 40 | 10 | 9 | Normoxia-NC |
|  |  | 50 | 10 | 10 | Normoxia-NC |
|  |  | 1 | 10 | 2 | Normoxia-KO1 |
|  |  | 10 | 10 | 3 | Normoxia-KO1 |
|  |  | 20 | 10 | 4 | Normoxia-KO1 |
|  |  | 30 | 10 | 4 | Normoxia-KO1 |
|  |  | 40 | 10 | 5 | Normoxia-KO1 |
|  |  | 50 | 10 | 6 | Normoxia-KO1 |
|  |  | 1 | 10 | 1 | Normoxia-KO2 |
|  |  | 10 | 10 | 2 | Normoxia-KO2 |
|  |  | 20 | 10 | 4 | Normoxia-KO2 |
|  |  | 30 | 10 | 5 | Normoxia-KO2 |
|  |  | 40 | 10 | 5 | Normoxia-KO2 |
|  |  | 50 | 10 | 6 | Normoxia-KO2 |
|  |  | 1 | 10 | 3 | Hypoxia-NC |
|  |  | 10 | 10 | 5 | Hypoxia-NC |
|  |  | 20 | 10 | 8 | Hypoxia-NC |
|  |  | 30 | 10 | 9 | Hypoxia-NC |
|  |  | 40 | 10 | 10 | Hypoxia-NC |
|  |  | 50 | 10 | 10 | Hypoxia-NC |
|  |  | 1 | 10 | 2 | Hypoxia-KO1 |
|  |  | 10 | 10 | 4 | Hypoxia-KO1 |
|  |  | 20 | 10 | 4 | Hypoxia-KO1 |
|  |  | 30 | 10 | 5 | Hypoxia-KO1 |
|  |  | 40 | 10 | 6 | Hypoxia-KO1 |
|  |  | 50 | 10 | 7 | Hypoxia-KO1 |
|  |  | 1 | 10 | 2 | Hypoxia-KO2 |
|  |  | 10 | 10 | 3 | Hypoxia-KO2 |
|  |  | 20 | 10 | 4 | Hypoxia-KO2 |
|  |  | 30 | 10 | 4 | Hypoxia-KO2 |
|  |  | 40 | 10 | 6 | Hypoxia-KO2 |
|  |  | 50 | 10 | 8 | Hypoxia-KO2 |
| Supplement Figure 3 | PN03-GSC | 1 | 10 | 1 | PLOD1-EV |
|  |  | 10 | 10 | 2 | PLOD1-EV |
|  |  | 20 | 10 | 5 | PLOD1-EV |
|  |  | 30 | 10 | 6 | PLOD1-EV |
|  |  | 40 | 10 | 7 | PLOD1-EV |
|  |  | 50 | 10 | 8 | PLOD1-EV |
|  |  | 1 | 10 | 4 | PLOD1-OE |
|  |  | 10 | 10 | 5 | PLOD1-OE |
|  |  | 20 | 10 | 6 | PLOD1-OE |
|  |  | 30 | 10 | 8 | PLOD1-OE |
|  |  | 40 | 10 | 9 | PLOD1-OE |
|  |  | 50 | 10 | 10 | PLOD1-OE |
|  | PN04-GSC | 1 | 10 | 2 | PLOD1-EV |
|  |  | 10 | 10 | 2 | PLOD1-EV |
|  |  | 20 | 10 | 4 | PLOD1-EV |
|  |  | 30 | 10 | 6 | PLOD1-EV |
|  |  | 40 | 10 | 7 | PLOD1-EV |
|  |  | 50 | 10 | 9 | PLOD1-EV |
|  |  | 1 | 10 | 4 | PLOD1-OE |
|  |  | 10 | 10 | 5 | PLOD1-OE |
|  |  | 20 | 10 | 7 | PLOD1-OE |
|  |  | 30 | 10 | 8 | PLOD1-OE |
|  |  | 40 | 10 | 9 | PLOD1-OE |
|  |  | 50 | 10 | 10 | PLOD1-OE |
