# Supplementary material for: Hypoxia-induced PLOD1 overexpression contributes to the malignant phenotype of glioblastoma via NF-κB signaling
Source: Oncogene. 2021 Jan 8;40(8):1458–75. doi: 10.1038/s41388-020-01635-y (PMC7906902; doi:10.1038/s41388-020-01635-y)
Supplement: Supplementary file 3 — Supplementary Table2 [file 41388_2020_1635_MOESM3_ESM.docx]

**Table S2.** Relationship of PLOD1 expression to clinical features of glioblastoma patients

| **Clinical features** | | **Samples**  **(*n* = 93)** | **PLOD1 expression** | | ***P* value** |
| --- | --- | --- | --- | --- | --- |
|  |  |  | **Low (*n* = 32)** | **High (*n* = 61)** |  |
| Sex | Male | 54 | 18 | 36 | 0.7973 |
|  | Female | 39 | 14 | 25 |  |
| Age | ≤ 50 | 57 | 15 | 42 | **0.0387** |
|  | > 50 | 36 | 17 | 19 |  |
| Subtype | proneural | 28 | 16 | 12 | **＜0.001** |
|  | classical | 30 | 12 | 18 |  |
|  | mesenchymal | 35 | 4 | 31 |  |

*: PLOD1 expression was detected by immunohistochemistry and evaluated according to the German immunohistochemical score. High expression was defined as score ≥4.
