# Supplementary material for: Hypoxia-induced PLOD1 overexpression contributes to the malignant phenotype of glioblastoma via NF-κB signaling
Source: Oncogene. 2021 Jan 8;40(8):1458–75. doi: 10.1038/s41388-020-01635-y (PMC7906902; doi:10.1038/s41388-020-01635-y)
Supplement: Supplementary file 4 — Supplementary Table3 [file 41388_2020_1635_MOESM4_ESM.docx]

Table S3. Clinical information of the primary glioma stem-like cells

|  | MES02-GSC | PN03-GSC | PN04-GSC | MES06-GSC | PN09-GSC | MES13-GSC |
| --- | --- | --- | --- | --- | --- | --- |
| Gender | Female | Female | Male | Male | Male | Female |
| Age | 78 years old | 54 years old | 66 years old | 68 years old | 43 years old | 39 years old |
| Location | Right frontal lobe | Left occipital lobe | Left occipital lobe | Left parietal lobe | Left insula | Right parietal lobe |
| Pathological | Glioblastoma | Glioblastoma | Glioblastoma | Glioblastoma | Glioblastoma | Glioblastoma |
| WHO grade | IV | IV | IV | IV | IV | IV |
| Ki-67 | 60% (+) | 20% (+) | 60% (+) | 60% (+) | 50% (+) | 60% (+) |
